# Building primary care providers' confidence in deprescribing opioids and benzodiazepines in older adults

**DOI:** 10.1016/j.rcsop.2025.100627

**Published:** 2025-06-24

**Authors:** Stefanie P. Ferreri, Lori T. Armistead, Ben Urick, Tamera D. Hughes, Anne-Therese Hunt, J. Marvin McBride, Joshua Niznik, Ellen Roberts, Kimberly A. Sanders, Jan Busby-Whitehead

**Affiliations:** a Division of Practice Advancement and Clinical Education, Eshelman School of Pharmacy, University of North Carolina, 115 Beard Hall, 301 Pharmacy Lane, Chapel Hill, NC 27599-7475, USA; b Hunt Consulting Associates, Chapel Hill, NC, USA; c Division of Geriatric Medicine and Center for Aging and Health, UNC School of Medicine, University of North Carolina, 5003 Old Clinic Building, Chapel Hill, NC 27599-7550, USA; d Division of Pharmaceutical Outcomes and Policy, Eshelman School of Pharmacy, 301 Pharmacy Lane, University of North Carolina, Chapel Hill, NC, USA

**Keywords:** Deprescribing, Primary care, Opioids, Benzodiazepines, Pharmacists, Potentially inappropriate medications

## Abstract

**Background:**

Opioids and benzodiazepines (BZDs) are among the most prescribed medications that contribute to falls in older adults; however, little guidance exists on their safe prescribing and deprescribing. Although some resources are available to assist providers with opioid and BZD deprescribing, many report lack of confidence as a barrier. The objective of this study was to assess PCPs' confidence in their ability to deprescribe opioids and BZDs before and after an intervention.

**Methods:**

We modified a validated deprescribing self-efficacy survey to assess primary care provider (PCP) confidence in deprescribing opioids and BZDs in older adults before and after a consultant pharmacist educational intervention. The survey consisted of 35 questions divided into three sections: deprescribing opioids (10 questions), deprescribing BZDs (10 questions), and deprescribing under potentially impeding circumstances [UPIC] (15 questions). The survey was sent to 88 PCPs using a modified Dillman method. We evaluated providers' confidence on a 100-point scale pre- and post-intervention, comparing the difference-in- differences (DID) in scores between the intervention and control groups.

**Results:**

A total of 41 PCPs (46.6 %) completed the survey both pre-and post-intervention. The intervention group (*n* = 21) showed an improvement in their knowledge and self-efficacy skills by an average of 19.7 out of 100 points, while the control group (*n* = 20) improved by an average of 5.2 points. The DID in self-efficacy improvement between the two groups was +14.5 points (*p* = 0.003) overall. For each of the opioid-, BZD-, and UPIC-specific scores, the intervention group had a statistically significant DID compared to the control group (+15.8, *p* = 0.004; +14.2, *p* = 0.017; +13.9, *p* = 0.016, respectively).

**Conclusion:**

This consultant pharmacist educational intervention improved PCPs' confidence in deprescribing opioids and BZDs in older adults.

## Highlights

• Chronic opioid and benzodiazepine (BZD) use increases older adults' falls risk.
• A consultant pharmacist educational intervention was provided to primary care providers (PCPs).
• A deprescribing self-efficacy survey was sent to PCPs pre- and post- intervention.
• The survey assessed self-efficacy in deprescribing opioids and BZDs in older adults.
• The PCPs' confidence in deprescribing opioids and BZDs in older adults improved.

## 1 Background

Inappropriate medication use among older adults poses risks of adverse medication events, injuries, hospitalization, and death.^1^, ^2^, ^3^, ^4^, ^5^, ^6^ To alleviate these risks, deprescribing inappropriate medications can be incorporated into primary care practices. Deprescribing is the planned and supervised process of dose reduction or stopping of a medication where the harm of the medication outweighs the benefit.^4^, ^5^, ^6^, 7., 8, 9 The goal of deprescribing is to reduce medication burden while improving the patient's quality of life.

As patients age, physiological changes occur that warrant deprescribing, especially for medications that carry a high risk for adverse effects.^3^, ^4^, ^5^ Many central nervous system (CNS)-active medications, such as opioids and benzodiazepines (BZDs), are considered potentially inappropriate medications for older adults due to an increased risk of cognitive impairment, falls, and falls-related injuries, particularly when taken at higher doses and/or in combination.^3^, ^4^, ^5^^,^^9^^,^^10^

Despite the need to reduce the inappropriate use of opioids and BZDs in older adults, there are many challenges providers face when attempting to deprescribe them.^4^^,^^9^^,^^11^, ^12^, ^13^, ^14^ These challenges may include a lack of suitable alternative therapies, potential for dependency, and fear of negative outcomes. ^4^^,^^9^^,^^12^, ^13^, ^14^ Many providers may be reluctant to start a conversation about deprescribing due to the time constraints of a patient visit.^11^^,^^13^^,^^14^ Providers may also have knowledge and/or skill deficits that lead to low confidence in their ability to successfully deprescribe these medications (low self-efficacy). ^4^^,^^11^^,^^13^^,^^14^ Lack of awareness of the importance of deprescribing, inertia (the failure to act despite awareness of potential issues), and devolvement of responsibility to other providers are other common barriers to deprescribing. ^9^^,^^13^^,^^14^ Inherent structural issues in the health care system itself – such as multiple providers prescribing medications for a patient, fragmented care, a culture of diagnosis and prescribing, and a lack of decision-making systems, tools, and resources – also contribute to the challenge. ^4^^,^^9^^,^^11^^,^^13^^,^^14^ Finally, patients themselves may provide barriers and may be resistant to deprescribing their opioids and/or BZDs due to unsuccessful prior experiences, physiological or psychological dependence on the medication(s), lack of trust in their provider, and/or fear of change. ^11^^,^^12^^,^^14^ Limited provider availability for follow-up, lack of patient understanding, and gaps in tailored support may also derail successful deprescribing efforts. ^4^^,^^11^, ^12^, ^13^, ^14^

The objective of this study was to assess PCPs' confidence in their ability to reduce unnecessary opioid and BZD use in their older adult patient populations before and after a deprescribing intervention.

## 2 Methods

This study was a subset of a larger randomized trial wherein primary care providers (PCPs) in North Carolina were part of a deprescribing intervention.^15^ The intervention included provider education materials (videos and website resources) as well as clinical support from consultant pharmacists. The pharmacists provided deprescribing recommendations in the electronic health record (EHR) communications.

### 2.1 Design

A validated deprescribing survey, described elsewhere,^16^ was modified to assess the confidence and self-efficacy of providers in 15 primary care clinics Surveys were distributed electronically to 88 providers, 50 (56.8 %) of whom were in the control group and 38 (43.2 %) of whom were in the intervention group. The pre-intervention surveys were sent between March 2020 and December 2020, and the post-intervention surveys were sent after the intervention concluded (between June 2021 and October 2021). Each provider had a month to complete the survey in the pre- and post- intervention periods. For the post-intervention survey, the providers received the survey within a month of intervention completion. Participants were given a gift card incentive for each survey (pre- and/or post-intervention) completed.

### 2.2 Materials

The deprescribing self-efficacy survey originally developed by Farrell et al. was adapted for use in this study.^16^ Changes to the survey instrument included creating an additional section focused on deprescribing opioids. Other changes to minimize respondent fatigue included revisions in the clinical role categories to better reflect the providers prescribing medications (i.e., elimination of the pharmacist category and addition of internist and physician assistant categories), elimination of the survey practice section, and reduction in the number of drug-specific sections (i.e., removal of proton pump inhibitors (PPIs), statins, and anti-psychotic drugs). Finally, the section on benzodiazepine receptor agonists (BZRA) was adjusted to cover only BZDs and two additional questions were added on determining whether using non-controlled medications would facilitate deprescribing opioids and BZDs. The final modified survey consisted of 35 questions divided into three sections focusing on provider self-efficacy in: deprescribing opioids (10 questions), deprescribing BZDs (10 questions), and deprescribing under potentially impeding circumstances [UPIC] (15 questions). Before using the modified instrument in this study, the reliability and validity of the adapted instrument was evaluated using a convenience sample of 22 primary care providers not affiliated with the study (22 % response rate). We used a cross-over design in which respondents were asked to complete the original and modified versions of the survey.^16^ The adapted instrument was found to meet criteria for both validity (construct and criterion) and reliability (internal consistency).

The survey questions used a Likert scale of 0–100 in 10-point increments, where a score of 0 indicated the provider felt they “cannot do [the task listed] at all”, a score of 50 indicated the provider felt they were “moderately certain they can do” the task listed, and a score of 100 indicated the provider felt they were “highly certain they can do” the task listed. An average composite score for each section was calculated to indicate prescriber confidence for that area (deprescribing opioids, deprescribing BZDs, and deprescribing UPIC). An overall average composite score was also tabulated to assess overall provider self-efficacy in deprescribing. See **Supplemental File S1** for a copy of the adapted survey instrument.

In the post-intervention survey, additional questions were asked of participants about what they liked and disliked about the intervention. Demographics such as clinical role (physician, nurse practitioner, physician assistant), years of experience working with patients ≥65 years old, sex, and age were also collected.

### 2.3 Analysis

The original validation paper from Farrell did not identify a threshold that would indicate a meaningful change. This was established based on statistical significance. Our study followed the same analysis. For the purposes of this study, only providers who completed both the pre- and post-intervention surveys were included in analysis. The mean scores for each of the three sections of the pre-intervention survey (opioids, BZDs, and UPIC) were compared to the mean scores of each of the three sections post-intervention (paired *t*-tests). This pre-post comparison was performed for both the intervention and control arms. The difference-in-differences (DID) for each set of scores (opioids, BZDs, and UPIC; intervention vs control) was calculated using a generalized linear model with a repeated measure for participant and fixed effects for period (i.e., pre- vs. post-intervention) and cohort (i.e., control vs. intervention). This study received Institutional Review Board (IRB) exemption.

## 3 Results

### 3.1 Respondent characteristics

A total of 60 providers (68.2 %) completed the pre-intervention survey (*n* = 28 intervention, *n* = 32 control) and 49 providers (55.7 %) completed the survey post-intervention (*n* = 24 intervention, *n* = 25 control). Forty-one providers (46.6 %) completed both the pre-intervention and post-intervention surveys (*n* = 21 intervention, *n* = 20 control). Of these 41 providers, the majority held an MD/DO degree (n = 24; 58.5 %), identified as female (n = 28; 68.3 %), and practiced 10 or more years (n = 24; 58.5 %). Table 1 highlights demographic information of the participating providers.

**Table 1:** Demographics of providers who completed pre- and post-intervention surveys.

	Total, N (%)	Intervention, N (%)	Control, N (%)
MD/DO	**24** (58.5)	**8** (38.1)	**16** (80.0)
NP	**9** (22.0)	**7** (33.3)	**2** (10.0)
PA	**8** (19.5)	**6** (28.6)	**2** (10.0)
Female	**28** (68.3)	**15** (71.4)	**13** (65.0)
Male	**13** (31.7)	**6** (28.6)	**7** (35.0)
34 and under	**5** (12.2)	**2** (9.5)	**3** (15.0)
35–44	**18** (43.9)	**10** (47.6)	**8** (50.0)
45–54	**12** (29.3)	**7** (33.3)	**5** (25.0)
55–64	**5** (12.2)	**1** (4.8)	**4** (20.0)
65 and older	**1** (2.4)	**1** (4.8)	**0** (0.0)
Fewer than 5 years	**5** (12.2)	**2** (9.5)	**3** (15.0)
5–9 years	**12** (29.3)	**7** (33.3)	**5** (25.0)
10–14 years	**8** (19.5)	**5** (23.8)	**3** (15.0)
15–19 years	**4** (9.8)	**0** (0.0)	**4** (20.0)
20–24 years	**7** (17.1)	**5** (23.8)	**2** (10.0)
25 years or more	**5** (12.2)	**2** (9.5)	**3** (15.0)
**Total**	**41**	**21**	**20**

Key: MD, medical doctor; DO, doctor of osteopathic medicine; NP, nurse practitioner; PA, physician assistant.

### 3.2 Pre-post comparisons

At baseline, the pre-intervention mean scores for self-efficacy appeared higher in the control. However, these differences were non-significant when examining the overlap in 95 % confidence intervals across groups. Statistically significant improvements in the intervention group's scores pre-to-post intervention were observed for the deprescribing opioids and deprescribing BZDs sections and for the overall average (pre-to-post differences: +21.8 for opioids; +21.3 for BZDs; +19.7 for overall average) (Table 2). Non-statistically significant improvements in pre-to-post scores were observed for the intervention group's UPIC scores and all control group scores.

**Table 2:** Self-efficacy scores for deprescribing pre- and post-intervention.

Outcome	Intervention			Control			Difference in Differences⁎	*p*-Value
	Pre, Mean N (95 % CI)	Post, Mean N (95 % CI)	Difference	Pre, Mean N (95 % CI)	Post, Mean N (95 % CI)	Difference		
Deprescribing Opioids	56.9 (47.5–66.2)	78.7 (70.1–87.2)	+21.8	65.3 (59.7–70.9)	71.3 (66.3–76.3)	+6.0	+15.8	0.004
Deprescribing Benzodiazepines	58.8 (48.9–68.6)	80.0 (70.9–89.2)	+21.3	63.5 (58.5–68.4)	70.6 (63.9–77.2)	+7.1	+14.2	0.017
Deprescribing under potentially impending circumstances (UPIC)	45.1 (35.9–54.4)	62.3 (52.3–72.4)	+17.2	52.7 (46.7–58.8)	56.1 (49.1–63.1)	+3.4	+13.9	0.016
Overall Average	52.4 (45.5–61.2)	72.1 (63.5–80.6)	+19.7	59.4 (54.3–64.5)	64.6 (59.6–69.5)	+5.2	+14.5	0.003

⁎ The difference-in-differences values were calculated by the difference in intervention scores (post-pre) minus the difference in control scores (post-pre). P-value is calculated for the period-by-group interaction.

### 3.3 DID comparison

**Fig. 1:**
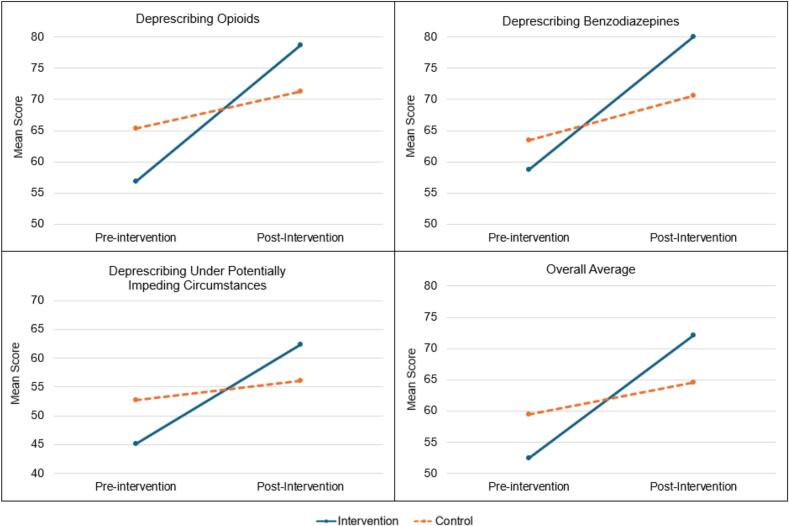
Difference in difference in deprescribing self-efficacy scores, intervention vs control.

Statistically significant DID between the intervention and control groups were observed when evaluating the change in deprescribing self-efficacy scores from pre- to post-intervention for opioids, BZDs, and UPIC. Table 2 and Fig. 1 compare the average deprescribing self-efficacy scores for the intervention and control groups pre- and post-intervention. The intervention group score increased an average of 15.8 points *more* than the control group's score pre-to-post intervention for opioids (*p* = 0.004). The DID scores for BZDs were + 14.2 (*p* = 0.017) and + 13.9 for UPIC (*p* = 0.016). When calculating the average across all three sections of the survey, the DID in overall deprescribing self-efficacy, intervention vs control, was +14.5 points (*p* = 0.003).

### 3.4 Qualitative intervention feedback

When participants were asked what they liked about the deprescribing intervention, responses varied regarding intervention resources. One participant stated, “The notes/recommendations were timely, patient-centered, and effective.” Another participant reported that “it was good to have a guide and resources to rely on for deprescribing.” Additional responses included participants having “increased awareness of opioid and BZD prescribing”, “alternatives to discuss,” “a plan with evidence and specialist (pharmacist) input,” a “detailed tapering schedule”, and “the guidelines for tapering.” Participants admitted that they did not use the educational materials (videos and website resources) very much, however those that did considered them to be of good quality. The participants appreciated the deprescribing recommendations provided by consultant pharmacists more. A full description of the providers' perceptions of the intervention has been provided elsewhere.^17^

## 4 Discussion

This study demonstrated there was an increase in mean deprescribing self-efficacy scores for both the intervention and control groups, pre-intervention to post-intervention. However, the increase in the intervention group's scores was significantly higher than the increase in scores for the control group. Additionally, the improvement in the intervention group's opioid- and BZD-specific section scores and their overall average score pre-to-post intervention were statistically significant, while none of the control group's increases in scores were. The statistically significant increase in the intervention group's scores combined with the statistically significant DID between intervention vs control group scores indicates that the providers receiving the educational intervention with patient-specific consultant pharmacist deprescribing recommendations increased in their confidence in deprescribing opioids and BZDs over the course of the intervention.

This study builds on prior work conducted by Farrell et al.^16^ Their intervention focused on implementing evidence-based guidelines on deprescribing PPIs, BZRAs, and antipsychotics in three long term care facilities and three family medicine practices over a 4–6-month period. This study focused on deprescribing BZDs and opioids in primary care clinics over an 8–12-month period with the support of consultant pharmacist's deprescribing recommendations and educational materials. In the Farrell study, the average provider self-efficacy scores increased 5.42 for PPIs, 2.53 for BZRAs, and 10.06 for antipsychotics. Only the group that received guidelines for antipsychotic deprescribing showed a statistically significant increase in self-efficacy.

This intervention resulted in a greater increase in mean provider self-efficacy scores pre-to-post intervention for the intervention group compared to the Farrell study (an increase of 17.2–21.8 points for this study vs 2.5–10.1 points for the Farrell study). However, a direct comparison cannot be made since the educational intervention in the Farrell study was a deprescribing guideline in long-term and primary care settings in Canada. This differs from our study where the educational intervention was clinical and included describing recommendations made by pharmacists in the EHR in primary care settings in North Carolina. Additionally, this study included a control group, allowing for a DID in self-efficacy between intervention and control groups, pre-to-post intervention, to be determined. Indeed, when comparing the intervention and control groups for this intervention, statistically significant DIDs in improvement of provider self-efficacy for opioids, BZDs, and under potentially impeding circumstances were all observed.

This deprescribing intervention, which included provider education and clinical support in the form of detailed consultant pharmacist deprescribing recommendations in the electronic health record (EHR) – significantly increased PCPs' confidence in broaching the subject of deprescribing opioids and BZDs with their patients and navigating the tapering process with patients who were willing to try.

When the providers were asked what they appreciated about the intervention, the following factors were mentioned: increased awareness about the opioids and BZDs their patients were taking, the educational aspects of the intervention, the specific and detailed recommendations (tapering schedules and alternative therapies), and the additional clinical support they received from the pharmacists. All these supports could in turn help providers convince patients that deprescribing should be considered for safety reasons.

However, supporting and educating providers in the art of deprescribing medications in a patient-centered manner is only one strategy that can be used to address the challenges associated with deprescribing. Additional strategies are needed for decreasing the barriers of *patient* hesitancy, not just improving providers' confidence and knowledge. For example, studies focusing on patient education have been successful as they allow patients to be more aware of the benefits and potential harms of specific medications and feel more engaged in their care.^18^, ^19^, ^20^ Therefore, a multi-pronged approach involving a combination of patient education, provider education, and clinical pharmacy support services will likely lead to the greatest success in deprescribing opioids and BZDs in older adults.

An institutional-based observational study of 82 healthcare providers employed in an Ethiopian hospital found the greatest factors influencing a provider's decision to deprescribe were: their education level, their years of experience, and the significance of the patients' health conditions.^21^ Quality of the patient-provider relationship was not a significant factor. They concluded that “a therapy-specific deprescribing algorithm, multidisciplinary collaboration [with clinical pharmacists], and continuous education development” should be used to help healthcare practitioners in the deprescribing process.^21^ These are the same factors that were appreciated by the provider participants in the intervention arm of this study.

### 4.1 Strengths and limitations

A key strength of this study was the use of a control group, thereby reducing the risk of bias in our analyses. Also, even though several providers only responded to either the pre- or the post-intervention surveys, the overall response rate for completion of both surveys was 46.6 %.A potential weakness was that this study modified and evaluated a validated survey instrument, however the new instrument was not validated. The modified survey met criteria for construct and criterion validity, as well as for reliability (internal consistency); however, it was not formally validated. Since the sample used for determining the reliability and validity of the survey was small (*n* = 22), the validity and reliability results should be considered preliminary.

Additionally, the control group providers were told about the study before its initiation, and they completed a pre-intervention survey. These factors may have increased their awareness of and attentiveness to opioid and BZD deprescribing in the subsequent months, thereby increasing their confidence in the topic – at least marginally, as evidenced by the slight increase in their self-efficacy scores pre-to-post intervention.

## 5 Conclusion

A pharmacist-led educational deprescribing intervention using educational resources and patient-specific consultant pharmacist deprescribing recommendations improved PCPs' confidence and self-efficacy in deprescribing opioids and BZDs for older adults in primary care practices.

## CRediT authorship contribution statement

**Stefanie P. Ferreri:** Writing – review & editing, Writing – original draft, Supervision, Methodology, Funding acquisition, Conceptualization. **Lori T. Armistead:** Writing – review & editing, Writing – original draft, Visualization, Methodology, Investigation, Formal analysis, Data curation, Conceptualization. **Ben Urick:** Methodology, Inversitigation, Formal analysis, Writing - review & editing. **Tamera D. Hughes:** Writing – review & editing, Methodology, Conceptualization. **Anne-Therese Hunt:** Writing – original draft, Formal analysis. **J. Marvin McBride:** Methodology, Conceptualization. **Joshua Niznik:** Writing – review & editing, Methodology, Conceptualization. **Ellen Roberts:** Writing – review & editing, Methodology, Conceptualization. **Kimberly A. Sanders:** Writing – review & editing, Conceptualization. **Jan Busby-Whitehead:** Writing – review & editing, Supervision, Methodology, Funding acquisition, Conceptualization.

## Declaration of generative AI and AI-assisted technologies in the writing process

During the preparation of this work the authors did not use generative AI or AI-assisted technologies in the writing process. The authors take(s) full responsibility for the content of the published article.

## Funding

This work was funded by the Centers for Disease Control and Prevention (CDC) under Cooperative Agreement (5U01CE002955). Dr. Niznik is supported by a career development award from the National Institutes on Aging (1K08AG071794). The findings and conclusions in this report are those of the authors and do not necessarily represent the official position of the Centers for Disease Control and Prevention.

## Declaration of competing interest

The authors declare that they have no known competing financial interests or personal relationships that could have appeared to influence the work reported in this paper.

## Appendix A Supplementary data

Supplementary material UNC Deprescribing Self Efficacy Survey Supplementary material

## References

[1] Klarin I., Wimo A., Fastbom J. (2005). The association of inappropriate drug use with hospitalisation and mortality: a population-based study of the very old. Drugs Aging.

[2] Lau D.T., Kasper J.D., Potter D.E., Lyles A., Bennett R.G. (2005). Hospitalization and death associated with potentially inappropriate medication prescriptions among elderly nursing home residents. Arch Intern Med.

[3] Atkin P.A., Veitch P.C., Veitch E.M., Ogle S.J. (1999). The epidemiology of serious adverse drug reactions among the elderly. Drugs Aging.

[4] Scott I.A., Hilmer S.N., Reeve E. (2015). Reducing inappropriate polypharmacy: the process of deprescribing. JAMA Intern Med.

[5] Fick D.M., Semla T.P., Steinman M. (2019). American Geriatrics Society 2019 updated AGS beers criteria® potentially inappropriate medication use in older adults. J Am Geriatr Soc.

[6] Reeve E., Shakib S., Hendrix I. (2014). Review of deprescribing processes and development of an evidence-based, patient-centred deprescribing process. Br J Clin Pharmacol.

[7] Bruy'`ere Research Institute (2019). What is deprescribing?. https://deprescribing.org/what-is-deprescribing/;.

[8] Opondo D., Eslami S., Visscher S. (2012). Inappropriateness of medication prescriptions to elderly patients in the primary care setting: a systematic review. PLoS One.

[9] Moriarty F., Thompson W., Boland F. (2022). Methods for evaluating the benefit and harms of deprescribing in observational research using routinely collected data. Res Soc Adm Pharm.

[10] Dowell D., Haegerich T.M., Chou R. (2016). CDC guideline for prescribing opioids for chronic pain - United States. MMWR Morb Mortal Wkly Report.

[11] Ailabouni N.J., Weir K.R., Reeve E. (2022). Barriers and enablers of older adults initiating a deprescribing conversation. Patient Educ Couns.

[12] Ribeiro P.R.S., Schlindwein A.D. (2021). Benzodiazepine deprescription strategies in chronic users: a systematic review. Fam Pract.

[13] Doherty A.J., Boland P., Reed J. (2020). Barriers and facilitators to deprescribing in primary care: a systematic review. BJGP Open.

[14] Anderson K., Stowasser D., Freeman C. (2014). Prescriber barriers and enablers to minimising potentially inappropriate medications in adults: a systematic review and thematic synthesis. BMJ Open.

[15] Niznik J., Ferreri S.P., Armistead L. (2022). A deprescribing medication program to evaluate falls in older adults: methods for a randomized pragmatic clinical trial. Trials.

[16] Farrell B., Richardson L., Raman-Wilms L. (2018). Self-efficacy for deprescribing: a survey for health care professionals using evidence-based deprescribing guidelines. Res Soc Adm Pharm.

[17] Gutierrez Euceda B., Ferreri S.P., Armistead L.T. (2023). A descriptive analysis of primary care providers’ interest in clinical pharmacy services. Explor Res Clin Soc Pharm.

[18] Niznik J.D., Collins B.J., Armistead L.T. (2021). Pharmacist interventions to deprescribe opioids and benzodiazepines in older adults: a rapid review. Res Soc Adm Pharm.

[19] Tannenbaum C., Martin P., Tamblyn R., Benedetti A., Ahmed S. (2014). Reduction of inappropriate benzodiazepine prescriptions among older adults through direct patient education: the EMPOWER cluster randomized trial. JAMA Intern Med.

[20] Martin P., Tamblyn R., Benedetti A. (2018). Effect of a pharmacist-led educational intervention on inappropriate medication prescriptions in older adults: the D-PRESCRIBE randomized clinical trial. JAMA.

[21] Tegegn H.G., Gebresillassseie B.M., Erku D.A. (2021 Sep). Deprescribing practice in a resource-limited setting: healthcare providers’ insights. Int J Clin Pract.

