# Supplementary material for: Building primary care providers' confidence in deprescribing opioids and benzodiazepines in older adults
Source: Explor Res Clin Soc Pharm. 2025 Jun 24;19:100627. doi: 10.1016/j.rcsop.2025.100627 (PMC12269610; doi:10.1016/j.rcsop.2025.100627)
Supplement: Supplementary material — UNC Deprescribing Self Efficacy Survey [file mmc1.docx]

S1. UNC Deprescribing Self Efficacy Survey

*Modified from: Farrell B, Richardson L, Raman-Wilms L, de Launay D, Alsabbagh MW, Conklin J. Self-efficacy for deprescribing: a survey for health care professionals using evidence-based deprescribing guidelines. Research in Social and Administrative Pharmacy. Published online: 2018 Jan;14(1):18-25.*

**Demographic Information**

What is your clinician role?

- Family Physician
- Geriatrician
- Internist
- Nurse Practitioner
- Physician Assistant
- Other: ________________________________________________

How many years of experience do you have working with patients over 65?

- Less than 5 years
- 5 - 9 years
- 10 - 14 years
- 15 - 19 years
- 20 - 24 years
- 25 years or more

Which of the following describes how you identify yourself?

- Male
- Female
- Other

What is your age?

- 34 and under
- 35 - 44
- 45 - 54
- 55 - 64
- 65 and older

**Deprescribing Self-Efficacy Survey**

This survey is designed to help us gain a better understanding of how clinicians rate their self-efficacy in deprescribing an elderly patient’s medication(s) and how that self-efficacy changes over time as clinicians use deprescribing guidelines. Self-efficacy refers to one’s belief in their capability to carry out specific tasks. In this case, we are interested in your belief in your capability to carry out the tasks related to deprescribing (tapering or stopping) a medication an elderly patient is currently taking.

**Class #1: Opioids**

Please rate how certain you are right now that you can carry out these tasks for deprescribing opioids by recording a number, 0 to 100 using the scale given below:

0 = cannot do at all; 50 = moderately certain can do; 100 = highly certain can do

[0, 10, 20, 30, 40, 50, 60, 70, 80, 90, 100]

For a patient 65 years of age and older who is taking an opioid, I am able to:

- Weigh the benefits vs. harms of continuing the opioid
- Weigh the benefits vs. harms of deprescribing the opioid
- Consider the patient's preferences, goals of therapy, and life expectancy in deciding whether to continue of deprescribe the opioid
- Determine whether a non-pharmacological intervention would facilitate deprescribing the opioid
- Determine whether using a non-controlled medication would facilitate deprescribing the opioid
- Determine the best dosing approach to deprescribing the opioid
- Develop a monitoring plan to determine the outcome of deprescribing the opioid
- Negotiate a deprescribing plan for the opioid with the patient and his/her caregivers
- Monitor and follow-up to determine the outcome of deprescribing the opioid
- Determine if opioid tapering should stop, or if the opioid should be restarted

**Class #2: Benzodiazepines**

Please rate how certain you are right now that you can carry out these tasks for deprescribing benzodiazepines (BZDs) by recording a number, 0 to 100 using the scale given below:

0 = cannot do at all; 50 = moderately certain can do; 100 = highly certain can do

[0, 10, 20, 30, 40, 50, 60, 70, 80, 90, 100]

For a patient 65 years of age and older who is taking a benzodiazepine (BZD), I am able to:

- Weigh the benefits vs. harms of continuing the BZD
- Weigh the benefits vs. harms of deprescribing the BZD
- Consider the patient’s preferences, goals of therapy and life expectancy in deciding whether to continue or deprescribe the BZD
- Determine whether a non-pharmacological intervention would facilitate deprescribing the BZD
- Determine whether using a non-controlled medication would facilitate deprescribing the BZD
- Determine the best dosing approach to deprescribing the BZD
- Develop a monitoring plan to determine the outcome of deprescribing the BZD
- Negotiate a deprescribing plan for the BZD with the patient and his/her caregivers
- Monitor and follow-up to determine the outcome of deprescribing the BZD
- Determine if BZD tapering should stop, or if the BZD should be restarted

|  |
| --- |

**Deprescribing under potentially impeding circumstances**

A number of situations are described below which can make it difficult to deprescribe medications in the elderly. Please rate how certain you are right now that you can deprescribe medications in the elderly by recording a number, 0 to 100 using the scale given below:

0 = cannot do at all; 50 = moderately certain can do; 100 = highly certain can do

[0, 10, 20, 30, 40, 50, 60, 70, 80, 90, 100]

For a patient 65 years of age and older, I am able to deprescribe a medication:

- When I am concerned about adverse drug withdrawal events
- When I am concerned about exacerbations of the underlying condition the drug is being used to treat
- When disease-specific clinical guidelines recommend the use of a medication
- When the medication is coupled to outcome metrics
- When I receive little support from colleagues for stopping or reducing medications
- When I have too much work to do
- When I am concerned about damage to my provider-patient relationship
- When the patient is resistant to change
- When the patient’s family/caregivers are resistant to change
- When there is no literature describing the effects of medication tapering or discontinuation
- When there is no guidance on how to taper or stop a medication
- When I am not the original prescriber of the medication
- When the medication was prescribed by a specialist
- When I am unsure why the medication was started originally
- When the medication is being used to treat an adverse effect of another medication
